# Cloning and characterization of an *Eimeria necatrix* gene encoding a gametocyte protein and associated with oocyst wall formation

**DOI:** 10.1186/1756-3305-7-27

**Published:** 2014-01-15

**Authors:** Dandan Liu, Liqin Cao, Yulan Zhu, Changjing Deng, Shijie Su, Jinjun Xu, Wenjie Jin, Jingui Li, Lili Wu, Jianping Tao

**Affiliations:** 1 Jiangsu Co-innovation Center for Prevention and Control of Important Animal Infectious Diseases and Zoonoses, Ministry of Education Key Lab for Avian Preventive Medicine, Key Lab of Jiangsu Preventive Veterinary Medicine, College of Veterinary Medicine, Yangzhou University, 12 East Wenhui Road, Yangzhou, Jiangsu 225009, People’s Republic of China

**Keywords:** *Eimeria*, Gametocyte protein, Gene, Cloning and expression, Immunolocalization

## Abstract

**Background:**

Gametocyte proteins of *Eimeria* (*E*.) spp. are important components of the oocyst wall and some have been used to develop transmission-blocking vaccines against avian coccidiosis.

**Methods:**

Total RNA isolated from *E. necatrix* gametocytes was utilized as templates for RT-PCR amplification and sequencing of cDNA encoding a gametocyte protein using gene-specific primers. The cDNA was cloned into the bacterial expression vector pET28a(+) and expressed in *E. coli* BL21 cells. The antigenicity of the recombinant gametocyte protein and its localization in different *E. necatrix* life-cycle stages were determined by western blot and indirect immunofluorescence analyses, respectively.

**Results:**

A 731-nucleotide sequence of cDNA [GenBank: KF649255] of *E. necatrix* had 97.7% identity to that of Et*gam22* of *E. tenella*. The cDNA ORF encoded a 186-amino acid protein containing a histidine-proline-rich region. The recombinant gametocyte protein (rEnGAM22) was predominately expressed in the insoluble inclusion body and recognized by antiserum from chickens immunized with oocysts of *E. necatrix*, *E. maxima* and *E. tenella*. A specific antibody to the rEnGAM22 protein recognized the wall-forming bodies in macrogametocytes and the walls of oocysts and sporocysts.

**Conclusions:**

The gene cloned from *E. necatrix* gametocytes is an ortholog to Et*gam22* of *E. tenella* and presents a potential target for future recombinant subunit vaccines against coccidiosis.

## Background

Coccidiosis is a parasitic disease of the intestinal tract of most domestic and wild animals and birds that is caused by protozoan parasites of the genus *Eimeria*. Seven *Eimeria* species (*E. acervulina*, *E. brunette*, *E. maxima, E. mitis, E. necatrix*, *E. praecox,* and *E. tenella*) are known to infect the intestinal tracts of chickens [1,2] and cause symptoms of coccidiosis, including weight loss, haemorrhagic diarrhea and death [3]. Although coccidiosis is a well-known disease, it still remains one of the most economically important parasitic diseases of the poultry industry worldwide. In China alone, annual costs of in-feed medications or live vaccines for the control of *Eimeria* are estimated to cost 30–60 million US dollars [4] and the global cost is probably in the excess of $800 million annually [5].

Prophylactic medications have been successfully used to control avian coccidiosis, but alternative strategies are needed due to the increasing emergence of drug-resistant parasites in commercial production settings [6]. The leading alternative to chemotherapeutic control is vaccination with live vaccines, which is dependent on vaccine-induced immune protection with oocysts comprising varied formulations of live wild-type or attenuated parasites of one or more species [7-9]. Moreover, there are several drawbacks to the use of live parasites, which include the need for cold storage, limited shelf-life of the vaccine, possible increased morbidity and mortality, and the risk of attenuated organisms reverting to a more pathogenic state. However subunit vaccines derived from intrinsic parasitic antigens or recombinant proteins from cloned DNA may overcome these difficulties [10].

Three gametocyte antigens (EmGAM56, EmGAM82, and EmGAM230) have been previously shown to play important roles in protection against *E. maxima* infections [11]. The subunit vaccine CoxAbix® was constructed with these proteins from purified gametocytes, and conveys transmission blocking immunity [12] that can reduce oocyst shedding. A previous field trial showed that it was at least as effective as the response from coccidiostat-fed broiler controls [13]. However, the purification of the gametocyte antigens is expensive, time-consuming, and laborious, because it relies on the affinity purification of the native gametocyte antigens from parasites. Hence, a substitute vaccine based on the recombinant forms of these proteins would be advantageous and is, therefore, the focus of the current research [13-15].

*E. necatrix* is a highly pathogenic coccidium and can cause high mortality in susceptible birds. The first and second generation meronts of *E. necatrix* are primarily located within in the mid-intestinal area of host chickens and later oocyst development occurs only in the caecum [16]. Coccidiosis caused by *E. necatrix* mainly occurs in chickens older than 8 weeks when raised on a litter floor [17,18].

Disease control relies exclusively on the protective immunity conferred to chickens. Therefore, to immunize chickens against *E. necatrix,* a planned immunization program with field isolates has been extensively implemented among breeder pullet flocks; nonetheless, such measures assumed risk of leading to outbreaks [19] and introducing pathogenic species into the environment. However, the development of subunit vaccines prepared from gametocyte antigens or recombinant proteins may overcome these difficulties. To the best of our knowledge, there are no previous reports regarding gametocyte antigens of *E. necatrix* and their genes. Therefore, the aim of the current study was to clone and identify a gametocyte antigen gene from *E. necatrix*, namely En*gam22*, according to cDNA sequences and localization characteristics of the recombinant protein within the gametocyte and oocyst wall, and to analyze the immunogenic characteristics of the recombinant protein.

## Methods

### Parasites and animals

The *E. necatrix* Yangzhou strain used in this study was isolated from chickens that died from *E. necatrix* infection in 2009 in Yangzhou, China, confirmed by microscopic examination and sequence analysis of the rRNA gene internal transcribed spacer (ITS) regions [20,21], and has been maintained in our laboratory. Oocysts were passaged by oral inoculation (5000 sporulated oocysts) to 3–4-week-old Suqiu Yellow chickens that were purchased on the day of hatching from the Poultry Institute, Chinese Academy of Agricultural Sciences (Yangzhou, China), reared in a coccidia-free isolation facility, and allowed unlimited access to water and food that contained no anticoccidial drugs or antibiotics. Feces were collected on post-infection (PI) days 7–12, and unsporulated and sporulated oocysts were purified by centrifugation, salt flotation, and treatment with sodium hypochlorite as previously described [22].

All animal care and procedures were conducted according to the guidelines for animal use in toxicology. The study protocol was approved by the Animal Care and Use Committee of the College of Veterinary Medicine, Yangzhou University.

### Gametocyte preparation

Gametocytes were isolated using previously published methods [11] with some slight modifications. Briefly, 5-week-old chickens were infected with 30 000 oocysts. At 168 h PI, the chickens were sacrificed and then guts removed and washed with cold SAC (1 mM phenylmethanesulfonyl fluoride, 1 mg/mL bovine serum albumin (BSA), 170 mM NaCl, 10 mM Tris–HCl pH 7, 10 mM glucose, and 5 mM CaCl_2_). The caeca were cut open and the mucosal tissues removed and incubated at 37°C in a beaker with 0.5 mg/mL of hyaluronidase in SAC. The digested mucosal tissues were filtered through a 17-μm mesh polymer filter and washed with SAC. The filtrate was then filtered through a 10-μm mesh once again, and the gametocytes accumulated on this filter were washed off with SAC and centrifuged at 3 000 rpm for 5 min and then stored at −80°C for future use.

### RNA extraction and amplification of the En*gam22* gene

Total RNA was isolated from purified gametocytes using TRIzol reagent (Invitrogen, Carlsbad, CA, USA) according to manufacturer’s instruction and then resuspended in diethylpyrocarbonate-treated water and was quantified using a UV spectrophotometer (NanoDrop2000; Thermo Fisher Scientific, Waltham, MA, USA) and stored at −80°C for further use. The sequence of the gene coding the gametocyte protein was amplified by reverse transcription polymerase chain reaction (RT-PCR) using the RNA LA PCR Kit (TaKaRa Bio. Inc., Shiga, Japan) following the manufacturer’s instruction. Specific primer sequences (Table 1, En1) were used at 0.2 mM each to amplify the target gene under the following conditions: an initial denaturation step at 94°C for 4 min; followed by 28 cycles of 94°C for 30 s, 56°C for 30 s, 72°C for 1.5 min, and a final elongation step at 72°C for 10 min. The PCR products were analyzed by 1.2% agarose gel electrophoresis.

**Table 1:** The primers used to amplify the target gene and coding sequence of EnGAM22

**Gene ID**	**Forward primer (5′-3′)**	**Reverse primer (5′-3′)**
En1	ACCCCAAAATAAAATCAAAGGC	CCATGAAGATCTCAGACGTAGC
En2	TCG*GAATTC*GACGGAGCACCTGAG	GCG*AAGCTT*TTAGTTGATGTCGGT

To confirm that the gene contained no intervening sequences, genomic DNA was isolated from purified gametocytes using the Universal Genomic DNA Extraction Kit ver. 3.0 (TaKaRa Bio., Inc.) following the manufacturer’s instructions, and then used as a template for PCR under the conditions described above.

### Cloning, sequencing, and DNA analyses

The RT-PCR products were purified and cloned into the TA vector pGEM-T-easy (Promega Corp., Madison, WI, USA) following the manufacturer’s instructions, which were then transformed into chemically competent DH5α *Escherichia coli* cells (Invitrogen). According to blue/white spot screening, white clones were selected for sequencing by a commercial sequencing company (Beijing Genomics Institute, Beijing, China). DNA sequences were analyzed using the BLASTN nucleotide alignment tool and predicted protein sequences were analyzed using DNAstar and the online ClustalW2 alignment tool (http://www.ebi.ac.uk/Tools/msa/clustalw2/).

### Recombinant protein expression and purification

Based on the DNA sequencing results, special primers (Table 1, En2) containing *Eco*R I and *Hin*d III restriction enzyme sites were designed to amplify the coding sequence of the gene excluding the signal peptide. Then, the amplicon was cloned into the bacterial expression vector pET28a(+) (Invitrogen). According to kanamycin-resistance selection, recombinant plasmids were verified by sequence analysis and transformed into chemically competent *E. coli* BL21 cells (Invitrogen).

Recombinant protein expression from *E. coli* BL21 cells grown in lysogeny broth (LB) medium containing 30 μg/mL kanamycin at 37°C was induced using 1 mM isopropyl β-D-1-thiogalactopyranoside (IPTG; Promega Corp.) at an absorbance at 600 nm of 0.6. The induced bacterial cells were incubated for 4 h and then harvested by centrifugation. The cell pellets were lysed in lysis equilibrium buffer (100 mM NaH_2_PO_4_, 10 mM Tris-Cl, 8 M urea, pH 8.0) and then sonicated (2 s/3 s, for 15 min). Next, the bacterial lysates were separated by sodium dodecyl sulfate polyacrylamide gel electrophoresis (SDS-PAGE) on 12% gels and visualized after staining with Coomassie brilliant blue. The recombinant 6 × His-tagged proteins were purified from the soluble fraction of the lysate using a Ni-NTA chromatography column (GenScript, Piscataway, NJ, USA) with 500 mM imidazole (Sigma-Aldrich, St. Louis, MO, USA). After the affinity-purified proteins were renatured in renaturation buffer (50 mM Tris–HCl, 0.15 M NaCl, pH 8.0) containing 6, 4, 2, or 1 M urea at 4°C for 8 h, respectively, they were further renatured in phosphate-buffered saline (PBS; pH 8.0) at 4°C for 8 h and concentrated using polyethylene glycol (PEG8000). The yield of the purified recombinant proteins was estimated using the NanoDrop2000 spectrophotometer (Thermo Fisher Scientific) at an absorbance at 280 nm. The purified recombinant protein (rEnGAM22) was visualized on 12% SDS-PAGE after staining with Coomassie brilliant blue, aliquoted, and stored at −20°C until further use.

### Generation of immune sera

Three groups of 10-day-old birds were orally inoculated with 2,000 sporulated oocysts of *E. necatrix*, *E. tenella,* and *E. maxima* and administered oral booster inoculations at 10 and 20 days later with of 2,500 sporulated oocysts, respectively, that were administered directly into the birds’ crops using a catheter. Blood samples were collected 7 days after the second booster. Serum was separated from the blood and stored at −80°C until further analyzed.

Mouse serum anti-rEnGAM22 was prepared as described previously [23,24]. Briefly, rEnGAM22 was diluted to 1 mg/mL in PBS and then emulsified with an equal volume of complete or incomplete Freund’s adjuvant (Sigma-Aldrich). Six-week-old BALB/c mice were immunized three times at 2-week intervals with 0.1 mg of rEnGAM22. Blood was collected 7 days after the second booster dose. Polyclonal mouse anti-rEnGAM22 antibody was separated from the blood and stored at −80°C until required. Antibody levels in the mouse sera samples were determined using an indirect enzyme-linked immunosorbent assay (ELISA) as described below.

### Immuno-blot analysis of rEnGAM22 and gametocyte extracts

rEnGAM22 was resolved over 12% SDS-PAGE and transferred onto nitrocellulose membranes for 2 h at 100 V [25]. After blocking with 3% BSA in tris-buffered saline (TBS) for 1 h at 37°C, the membranes were incubated with anti-6 × His tag monoclonal antibody (dilution, 1:500; BBI Solutions, Cardiff, UK), mouse anti-rEnGAM22 polyclonal antibody (dilution, 1:100) or the convalescent chicken sera (dilution, 1:50) at room temperature for 1 h prior to washing three times with 0.03% Tween-20/TBS (TBST) for 10 min, respectively. The membrane-bound antibodies were then detected using horseradish peroxidase (HRP)-conjugated rabbit anti-chicken immunoglobulin G (IgG; dilution, 1:1000; GenScript) or HRP-conjugated goat anti-mouse IgG (dilution, 1:5000; Kirkegaard & Perry Laboratories, Inc. (KPL), Gathersburg, MD, USA), respectively, and developed in the presence of o-Phenylenediamine dihydrochloride (OPD) (Sigma-Aldrich) after washing five times with TBST for 10 min, respectively. Naïve sera from chicken and mice were used as a negative controls.

To confirm that the localization of EnGAM22 in the subsequent experiment was indeed only that of EnGAM22 and not in part due to cross-reactivity with higher- weight gametocyte proteins such as GAM56- or GAM82-like proteins, the gametocyte extracts were prepared following previously published techniques [26], and were used for immunoblot analysis under the conditions described above.

### Preparation of tissue samples and indirect immunofluorescence analysis

Preparation of tissue samples and indirect immunofluorescence analysis were performed as described previously [23,24]. Briefly, chickens were orally infected with 30,000 *E. necatrix* sporulated oocysts and sacrificed by CO_2_ inhalation and cervical dislocation at 132, 144, 156, 168, 180 and 192 h PI, respectively. The caeca and small intestines at 132 h PI were removed and fixed in 3% paraformaldehyde in PBS, respectively. Fixed tissues were embedded in paraffin, and then cut into 5 μm- thick sections using a microtome at room temperature. The paraffin was removed from the sections prior to inactivation of endogenous enzymes with 3% H_2_O_2_ and antigen retrieval using 0.1% trypsin (Promega Corp.). After blocking overnight in 5% BSA in PBS (BSA/PBS) at 4°C in a humidified chamber, the sections were incubated with mouse anti-EnGAM22 antibody (dilution, 1:100) in BSA/PBS for 1 h at 37°C, then washed in 0.03% TWEEN-20/PBS (PBST) three times for 15 min. Next, the sections were incubated with fluorescein isothiocyanate (FITC)-conjugate goat anti-mouse antibody (dilution, 1:100; KPL) in BSA/PBS for 1 h at 37°C and then rinsed in PBST as described above. The sections were then counter-stained with 4′,6-diamidino-2-phenylindole (DAPI; Roche Applied Science, Penzberg, Germany) for 5 min prior to mounting under coverslips with FluorSave Reagent (Bioworld, Consulting Laboratories, Mt. Airy, MD, USA) for visualization. Images were obtained using a Leica DM2500 reflected fluorescence microscope (Leica Microsystems GmbH, Wetzlar, Germany). Portions of the same paraffin-embedded tissue samples were also stained with hematoxylin and eosin to confirm the gametocyte and oocyst developmental processes in caecum mucosal tissues.

### Preparation of oocyst walls and sporocysts and indirect immunofluorescence analysis

The purified oocyst walls and sporocysts were prepared [24,27,28] and indirect immunofluorescence analyses of the same were performed as described previously [29,30]. Briefly, the sporulated oocysts (1 × 10^7^) were washed in distilled water (2,500 rpm, 15 min, 4°C) three times to remove the 2% potassium dichromate storage solution and then resuspended in five volumes of PBS and sonicated with an output 3.0, duty cycle 30% for 3 s intervals over 4 min in an icewater bath, until about 95% of the oocyst walls were broken. The sonicate fractions containing oocyst walls and sporocysts were centrifuged at 4,000 rpm for 10 min and the subsequent pellets were collected, washed with PBS for three times resuspended in 20 volumes of 1.1 M sucrose and centrifuged at 2,500 rpm for 15 min. The top layer of fluid (sporocysts) and the pellet (oocyst walls) were collected and washed with PBS for three times, respectively. The oocyst walls and sporocysts were resuspended in methanol (−20°C) and coated on glass slides, incubated for 10 min at −20°C and washed three times with PBS for 15 min each prior to submersion in 0.1% Triton X-100 in PBS for 10 min. After washing with PBS, the slides were blocked with 3% BSA/PBS and then stained with mouse anti-rEnGAM22 antibodies (dilution, 1:50). Lastly, the samples were labeled with tetramethyl rhodamin isothiocyanate (TRITC)-conjugated goat anti-mouse antibodies dissolved in BSA/PBS (dilution, 1:100) and imaged as described above.

## Results

### En*gam22* isolation and sequence analysis

**Figure 1:**
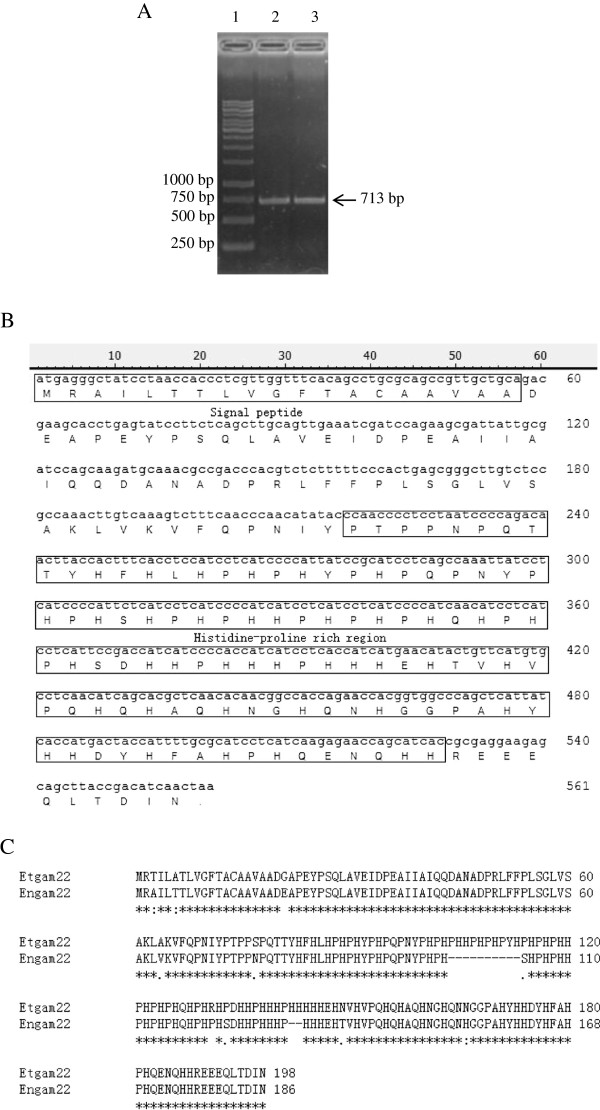
**Sequence analysis of En*gam22*. (A)** RT-PCR product of Engam22. Total RNA was extracted from purified gametocytes at 168 h PI, target gene was amplified using specific primer En1 (Table 1). Lane 1 was DNA marker, lane 2 and 3 were products of En*gam22*. **(B)** Predicted protein sequence of En*gam22.* The signal peptide and histidine-proline rich region was marked by box, and the splice site was between 19 and 20 amino acid. **(C)** Comparison of GAM22 proteins from *E. tenella* and *E. necatrix*. Invariable amino acid positions are marked with asterisks, and substitutions rated conservative and semiconservative by ClustalW are marked with colons and periods, respectively.

The phylogenetic tree analysis of small-subunit ribosomal RNA gene showed that *E. necatrix* and *E. tenella* were most closely related among the known species of coccidia (Sporozoa) [31]. Therefore, according to the *E. tenella* gametocyte protein gene sequence (Et*gam22*; GenBank accession number: CS000361), a pair of primers (Table 1, En1) was designed to amplify the *E. necatrix* gametocyte protein gene of interest, En*gam22*. The RT-PCR amplification product is depicted in Figure 1A. The sequence obtained for the En*gam22* cDNA comprised 713 base pairs (bp) (GenBank: KF649255) that included a single 561 bp open reading frame (ORF; Figure 1A), which had 97.7% identity to the 597-bp sequence of Et*gam22*. The En*gam22* cDNA ORF encodes a 186-amino acid (aa) polypeptide containing a histidine-proline-rich region (aa 73–176) that has been implicated in oocyst wall formation in other *Eimeria* species (Figure 1B). A comparison of the aa sequences of EnGAM22 and EtGAM22 via ClustalW multiple sequence alignment is shown in Figure 1C. As shown, the aa composition of the protein deduced from En*gam22*, like Et*gam22*, is very unusual, in that only four aa [His (22.0%), Pro (15.6%), Gln (7.5%) and Ala (8.6%)], accounted for nearly 54% of all residues. EnGAM22 has a 12 aa deletion within the histidine-proline-rich region and a very high aa identity (94.7%) to EtGAM22. Analysis using the SignalP program (http://www.cbs.dtu.dk/services/SignalP/) revealed that the N-terminus of EnGAM22 contained a 19-aa signal peptide (Figure 1B). The mature protein was predicted to be 21.39 kDa with a pI of 6.73. Comparatively, in an analysis using cDNA isolated from gametocytes, amplification of genomic DNA gave rise to a fragment of the same size, indicating that the gene contained no introns.

### rEnGAM22 expression and purification

**Figure 2:**
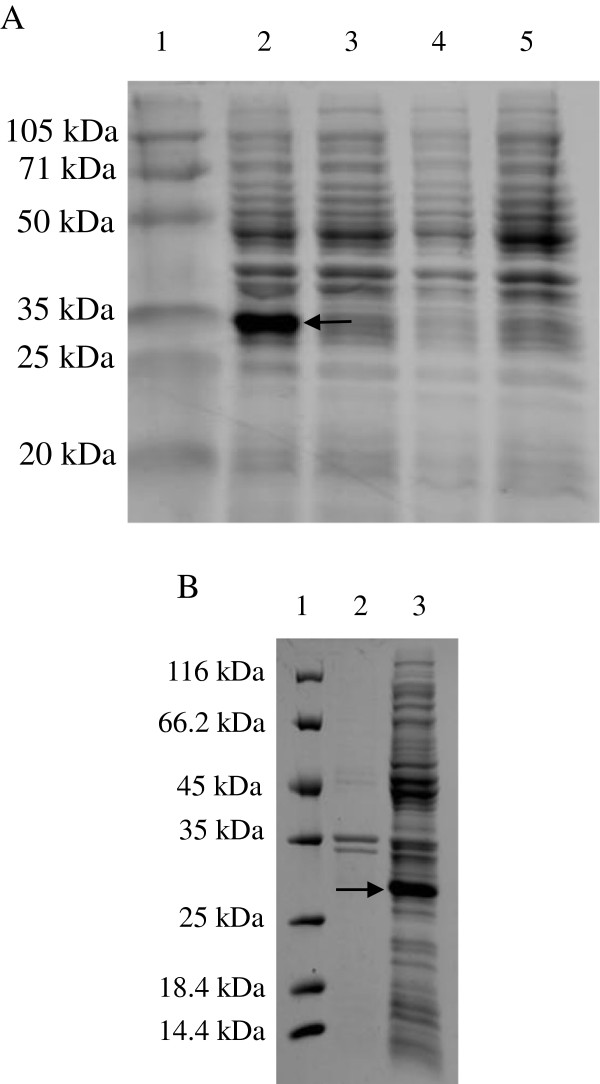
**Expression of recombinant gametocyte protein EnGAM22. (A)** Analysis of the expressed protein by SDS-PAGE. Protein marker (lane 1), the recombinant bacterial BL21 containing recombinant expression vector induced by 1 mM IPTG (lane 2), the recombinant bacterial BL21 not induced by IPTG (lane3), the bacterial containing the wild type vector induced by IPTG (lane 4) and the bacterial BL21 induced by IPTG (lane 5). **(B)** The solubility analysis of recombinant protein. Protein marker (lane 1), supernatant of bacterial sonicates (lane 2) and sediments of bacterial sonicates (lane 3).

A PCR product of ~522 bp was isolated from an agarose gel and subcloned into a pET28a(+) bacterial expression vector containing the NH_2_ terminal 6 × His tag prior to transformation into chemically competent *E. coli* BL21 cells. After the bacteria containing the expression vector were induced with 1 mM IPTG for 4 h at 37°C, the recombinant proteins expressed in the bacterial lysates were subjected to 12% SDS-PAGE, which revealed a protein band of ~29 kDa after staining with Coomassie brilliant blue (Figure 2A, lane 2) that had migrated less far than the expected 23.3 kDa recombinant protein. A band of the target protein was not detected in the bacterial lysates that had not been induced with IPTG (Figure 2A, lane 3). Similarly, the protein was not detected in either the bacteria containing the wild-type vector (Figure 2A, lane 4) or the control bacteria (Figure 2A, lane 5). The recombinant protein was mostly insoluble (Figure 2B) and, therefore, was purified from the soluble fraction of the bacterial lysates using a Ni-NTA chromatography column. After being concentrated with PEG8000, the final protein concentration was 10 mg/L.

### Antibody quantification by ELISA

The ELISA protocol was essentially the same as that described previously [32]. Briefly, rEnGAM22 was diluted in 50 mM carbonate buffer (pH 9.6) and 1 μg/well was coated into 96-well microtiter plates, incubated at 4°C overnight, then washed with PBS (pH 8.0) three times for 5 min and blocked with 1% BSA for 1 h at 37°C prior to incubation with PBS-diluted mouse sera for 1 h at 37°C. After washing three times for 5 min with PBST, HRP-conjugated goat anti-mouse IgG (dilution, 1:5000; KPL) was added to each well. After incubation for 60 min at 37°C the plates were washed five times with PBST for 5 min. Finally, immune complexes were revealed by incubating with tetramethylbenzidine (TMB, Sigma-Aldrich) and 0.3% H_2_O_2_ for 10 min. The reaction was stopped by adding 2 M H_2_SO_4_ and the absorbance values was read at 450 nm using an automatic MicroELISA reader (Sunrise-Basic; Tecan Trading AG, Männedorf, Switzerland). All samples were run in triplicate. The results revealed a relatively high rEnGAM22-induced antibody level in the experimental mice. The optical density (OD) values were as high as 2.15 using diluted (1:200) immunized mouse serum, but only 0.15 with the control mouse serum.

### Immunoblot analysis of rEnGAM22 and the gametocyte extracts

**Figure 3:**
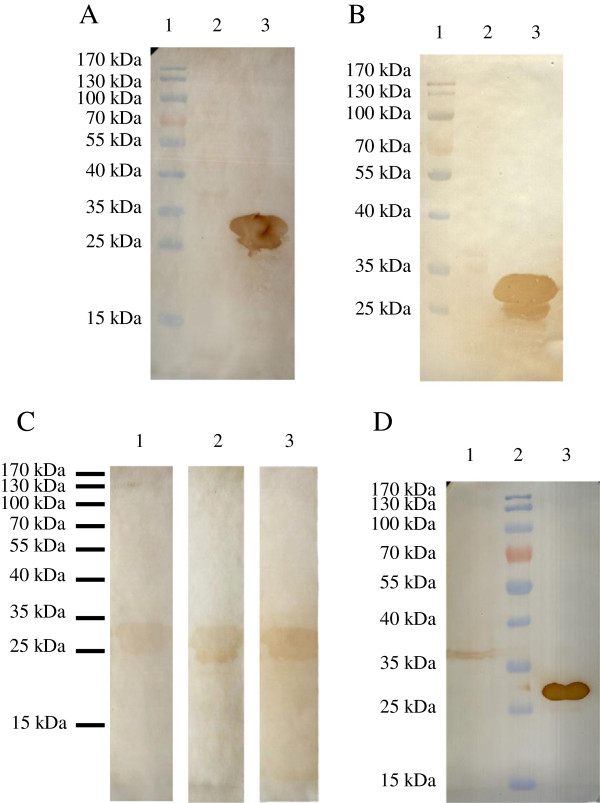
**Western blotting analysis of rEnGAM22.** The expressed protein was separated by SDS-PAGE and transferred to NC membranes, at the same time the wild type vector transferred bacterial was used as control. In **(A)** and **(B)**, anti-6 × His tag monoclonal antibody **(A)** and mouse anti-GAM22 polyclonal antibody **(B)** were used as first antibody, individually. Protein marker (lane 1), the control (lane 2) and the recombinant protein (lane 3). **(C)** The convalescent sera from chickens were used as first antibody, anti-*E. necatrix* (lane 1), anti-*E. tenella* (lane 2) and anti-*E. maxima* (lane 3). **(D)** The gametocyte extracts were detected by the mouse anti-rEnGAM22 antibody. Gametocyte extracts (lane 1), protein marker (lane 2), and purified rEnGAM22 (lane 3).

When immunoblots of the purified protein were probed using the anti-6 × His epitope tag monoclonal antibody (Figure 3A), apart from the expected 29 kDa band, an additional 25 kDa band was detected, which may have represented breakdown products of the full-length protein [13]. Similarly, two bands were detected using the mouse anti-rEnGAM22 antibody (Figure 3B) and the convalescent chicken sera (Figure 3C), respectively. However, among the gametocyte extracts, only a protein band of ~ 36 kDa was detected with the mouse anti-rEnGAM22 antibody, migrated less far than that of rEnGAM22 (Figure 3D). These bands were not detected when the control mouse or chicken sera control samples were used (data not shown).

### rEnGAM22 antibodies localized to macrogametes and developing oocyst in *E. necatrix*

Analysis of En*gam22* revealed a 247–528 bp nucleotide sequence encoding a histidine-proline-rich region, which has been previously implicated in oocyst wall formation in *E. tenella*[30]. To determine whether the EnGAM22 also played a role in oocyst wall formation, we used the mouse anti-rEnGAM22 polyclonal antibody to localize the EnGAM22 during different *E. necatrix* life-cycles stages.

**Figure 4:**
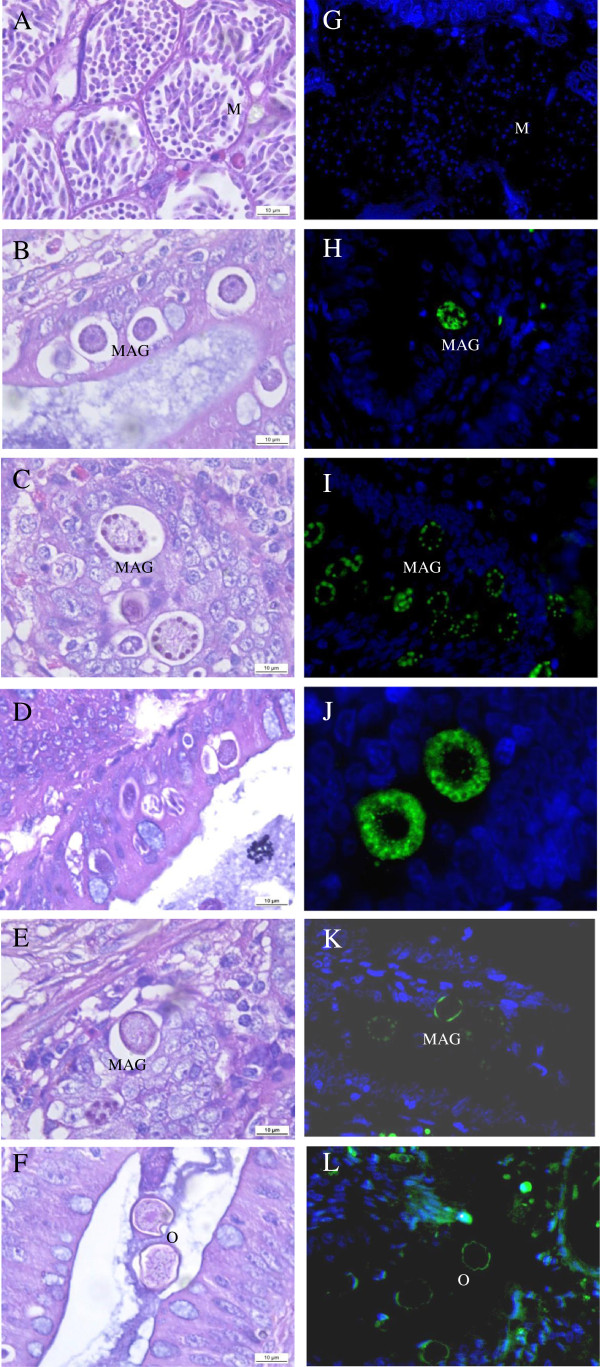
**Localization of the anti-rEnGAM22 antibodies to the endogenous developmental stages of *E. necatrix*.** The micrographs of endogenous stages of *E. necatrix* in histological sections, stained with H&E **(A-F)** and immuno-stained with anti-rEnGAM22 and visualised with FITC (green) and counter stained with DAPI (blue) **(G-L)**. Abbreviations: M, mature meront; MAG, macrogamont; O, oocyst. Bar represents 10 μm, or images photographed at × 1 000 magnification. **(A, G)** Meronts with distinct merozoites in the crypt epithelial cells of the midintestinal area at 132 h PI. **(B, H)** Mid-stage macrogametocytes in the crypt epithelial cells of the caeca at 156 h PI. **(C, I)** Mature macrogametes at 168 h PI. **(J)** Enlargement of macrogametes of Figure 4I. **(D)** The third generation mature meront at 168 h PI. **(E, K)** A macrogamete to initiate the formation of the first layer of the wall at 168 h PI. **(F, L)** Mature oocysts located in cecal contents at 168 h PI.

Second and third generation meronts, all stages of microgametocyte and macrogametocyte development plus oocyst formation, were observed in the haematoxylin and eosin stained tissue sections. However, parasitic development was asynchronous. At 132 h PI, the second generation of mature meronts contained a relatively high number of fully developed visible merozoites in the crypt epithelial cells retrieved from the mid-intestinal area (Figure 4A). At the same time point, the trophozoites or early gametes appeared in the epithelial cells of the caeca. At 156 h PI, a relatively large number of developing macrogametocytes were present in the lamina propria of the caeca (Figure 4B). At 168 h PI, most macrogametocytes developed into mature macrogametes (Figure 4C) and some exhibited formation of the first wall layer (Figure 4E) or appeared as oocysts (Figure 4F). At 192 h PI, a large number of mature oocysts were detected among the epithelial cells layers and within the cecal contents. The third generation of mature meronts was detected in all sections at 144–192 h PI (Figure 4D).

The parasites at the same developmental stage were visible in histological sections immunostained with anti-rEnGAM22 polyclonal mouse serum and visualized with FITC (green) and counter-stained with DAPI (blue). The anti-rEnGAM22 antibody localized to the wall forming bodies of the macrogametocytes (Figure 4H, I, J) and the oocyst walls (Figure 4K, L). The anti-rEnGAM22 antibody seemed to be localised to the wall forming body type 2 (Figure 4H, J). However, the microgametes and merozoites were not recognized by the anti-rEnGAM22 antibody (Figure 4G), indicating that the EnGAM22 protein is not expressed during the schizogonic stage. In all cases, staining of the anti-rEnGAM22 polyclonal mouse serum was not observed when the tissue sections were probed with normal mouse serum.

### Antibodies to rEnGAM22 localized to oocyst and sporocyst walls in *E. necatrix*

**Figure 5:**
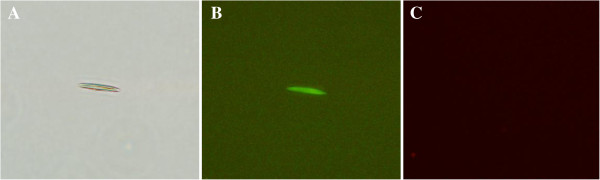
**UV autofluorescence detection of *E. necatrix *oocyst walls. (A)** Oocyst wall visualized under bright-field. **(B)** The same oocyst wall visualized under 330- to 385-nm UV light. **(C)** The same oocyst wall visualized under 520- to 550-nm light, which could not be detected by red fluorescence. Images photographed at × 1 000 magnification.

**Figure 6:**
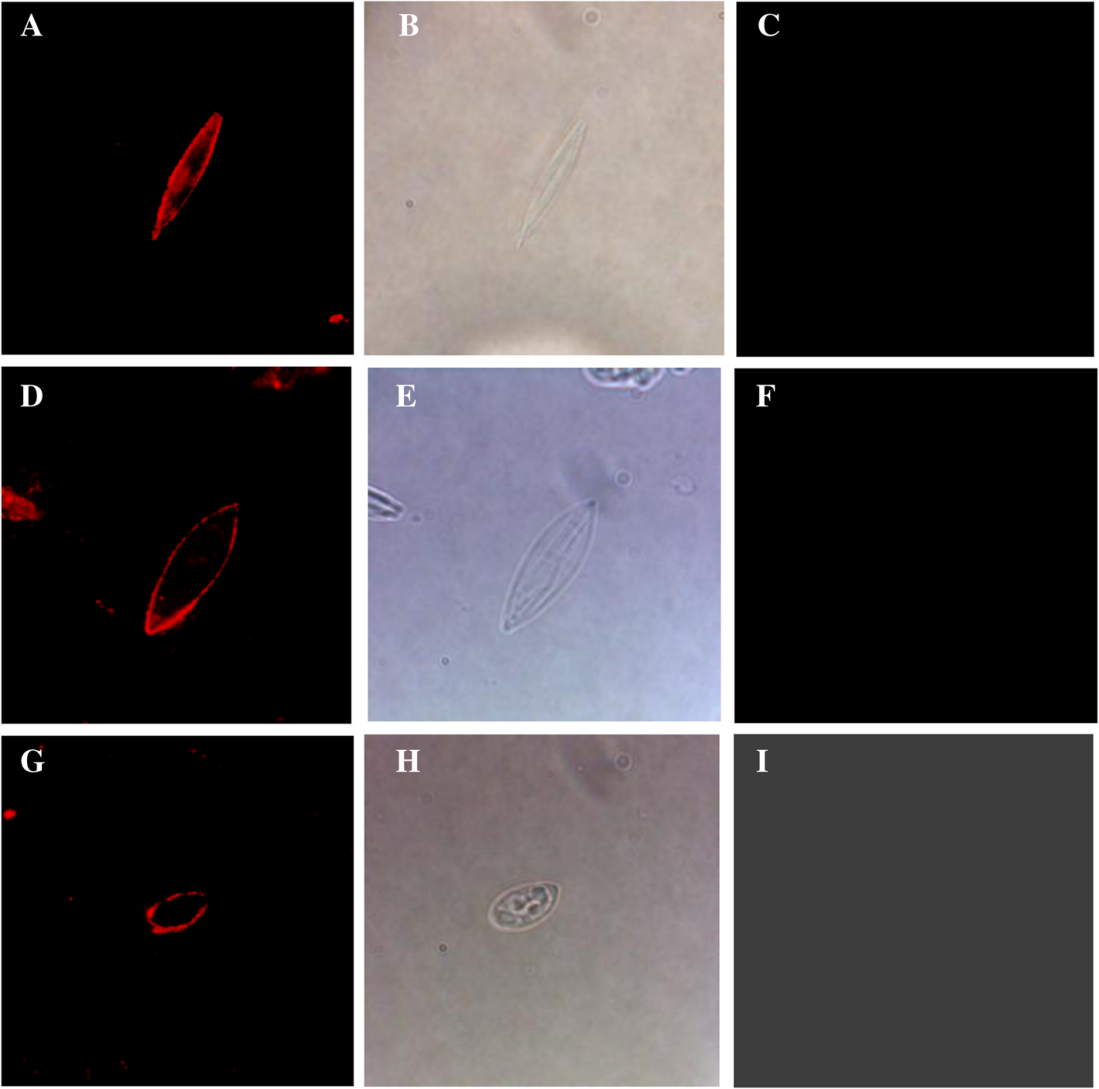
**Anti-EnGAM22 localize to the oocyst walls and sporocyst walls of *E. necatrix*.** Anti-EnGAM22 recognition of unsporolated oocyst walls **(Panel A)**, sporolated oocyst walls **(Panel D)** and sporocyst outer walls **(Panel G)** was represented. Bright-field images of unsporolated oocyst walls **(Panel B)**, sporolated oocyst walls **(Panel E)** and sporocyst outer walls **(Panel H)** were also represented. As a control, the negative control antibody could not recognize unsporolated oocyst walls **(Panel C)**, sporolated oocyst walls **(Panel F)** and sporocyst outer walls **(Panel I)**. Images photographed at × 1 000 magnification.

Since the oocyst walls autofluoresced blue (Figure 5), the oocyst walls and sporocysts immunostained with anti-rEnGAM22 antibody were visualized with TRITC in this experiment. The anti-rEnGAM22 antibodies were localized to the unsporolated oocyst walls (Figure 6A), the sporolated oocyst walls (Figure 6D), and the outer sporocyst walls (Figure 6G). However, these parasitic stages were not recognized by the negative control antibody (Figure 6C, F, I).

## Discussion

The coccidian oocyst wall is a bilayered structure formed from the contents of two specific organelles, types 1 and 2 wall forming bodies (WFB1 and WFB2, respectively), found exclusively in the macrogametes stage of coccidian parasites [33,34]. The oocyst wall consists mainly of proteins and lipids [27,28,34]. Gametocyte proteins are precursors of the oocyst wall proteins [28,34]. To date, only a small number of genes encoding gametocyte proteins have been cloned and sequenced from avian *Eimeria* species, such as Em*gam56*[25], Em*gam82*[24], and Em*gam230* (a partial sequence) [35] in *E. maxima*, Et*gam56* (Et*gam56 tmp 1*), Et*gam59* (Et*gam56 tmp 2*) and Et*gam22* in *E. tenella*[30,36], and Ea*gam56* in *E. acervulina* (a partial sequence) [36]. Other than Et*gam22,* which predicts a His- and Pro-rich domain [30], all analyses of Em*gam56*, Et*gam56,* Et*gam59,* and Em*gam82* have predicted two distinct protein domains [25,29,30,36]. Moreover, all of them are expressed specifically in gametocytes, although gametocyte-specific expression has not been detected in Et*gam59*[30]. Therefore, the features of sequence characteristics and gametocyte-specific expression can be used to clone and identify the genes encoding gametocyte proteins in *Eimeria* species.

In the present study, we cloned a novel gene from both genomic DNA and cDNA isolated from *E. necatrix* gametocytes and found that the nucleotide sequences were comprised of 731 bp with a 561-bp ORF encoding a 186-aa protein. The protein deduced from the gene had a His- and Pro-rich domain. The antibody prepared against the recombinant protein expressed in *E. coli* BL21 cells recognized the wall-forming bodies in macrogametocytes, the walls of oocysts and sporocysts, but did not recognize either microgametes or the merozoites. In the gametocyte extracts, the anti-rEnGAM22 antibody recognized a ~36 kDa protein representing EnGAM22. These results were consistent with previous findings regarding Et*gam22*[30], and further confirmed that the gene is an *E. necatrix* ortholog of Et*gam22* of *E. tenella*.

A previous study reported that both the 56 and 82 kDa gametocyte proteins in *E. maxima*, with true masses of 52.45 and 62.45 kDa [25], failed to migrate true-to-size by SDS-PAGE; particularly, the 82 kDa protein due to its unusual aa composition [24]. Similarly, in bacterial lysates expressing the r82 construct, a 75 kDa band was detected, which migrated further than that expected of a similar recombinant protein of an expected size of 67 kDa [13]. In the present study, both the recombinant and native EnGAM22 proteins failed to migrate true-to-size by SDS-PAGE, as the bands migrated further than that expected by theoretical molecular weights. The reason for this observation was likely due to an unusual aa composition, as in EmGAM82. Additionally, in vitro translation of Et*gam*22 cDNA produced a band of ~ 25 kDa by SDS-PAGE, which also migrated less far than that expected according to the theoretical molecular weight of 22.8 kDa [30].

Unlike Em*gam56*, Et*gam56,* Et*gam59* and Em*gam82* that are single-copy intron-free genes [29,30,36,37], Et*gam22* is an intron-free multicopy gene with ~12 –22 copies in head-to-tail arrangement [30]. Although Et*gam22* mRNA was readily detectable in the cecal tissue samples taken from *E. tenella*-infected chickens at 137 h PI, the EtGAM22 protein only became detectable at 168 h PI using the E2E5 antibody via Western blot analysis when the first unsporulated oocysts appeared in the cecum [30]. En*gam22*, like Et*gam22*, is expressed specifically at the gametocyte stage. However, the immunolocalisation results in different *E. necatrix* life-cycle stages revealed that En*gam22* expression may occur in the mid-stage of gametocyte development (at 156 h PI), and EnGAM22 is transported into WFB2 prior to participation in the formation of the inner oocyst wall. The possible explanations for this difference may be due to the methods to detect the expression products. Furthermore, similar to Et*gam22*, En*gam22* can be cloned directly from genomic DNA, implying En*gam22* is an intron-free gene. Thus, further studies are warranted to determine whether *E. necatrix* En*gam22* is indeed a multicopy gene.

Previous reports have confirmed that *Eimeria* gametocyte proteins are highly immunogenic [11,26,38,39]. Vaccination with gametocyte proteins of *Eimeria* species induces production of immunoprotective antibodies in breeding hens, which are then transferred to the developing embryos via the egg yolk, providing partial immunity to chicks upon hatching [13,40,41]. The anti-EmAPGA (antibody to *E. maxima* affinity purified gametocyte antigens) recognized proteins within the WFBs of macrogametocytes and oocyst walls of *E. maxima*, *E. tenella* and *E. acervulina*[36], which explained the features for the vaccine (CoxAbic®) against infections of *E. maxima* and heterologous species such *E. acervulina* and *E. tenella*[26]. The antibodies anti-rEmGAM56 and anti-rEmGAM82 raised against recombinant *E. maxima* gametocyte proteins, like anti-EmAPGA, reacted with various-sized proteins of gametocyte and oocyst preparations from *E. maxima*, *E. tenella,* and *E. acervulina*[36], in spite of only recognizing the WFB2 and inner oocyst wall via immunolocalisation analysis [33,36]. Gametocyte antigens of both *E. maxima* recombinant GAM56 and GAM82 were recognized by protective chicken serum raised against APGA, and can elicit a dose-dependent antibody response in chickens, suggesting that the recombinant antigens maintain the antigenic and immunogenic properties of the native proteins [13]. Furthermore, the recombinant GAM82 gametocyte antigen may stimulate the production of antigen-specific serum antibodies and a higher level of IL-2 and IL-15 mRNA, and induce protective intestinal immunity resulting in decreased oocysts shedding and reduced gut pathology [14]. In the present study, an analysis was also performed to determine whether the recombinant protein rEnGAM22 was recognized by sera from chickens that had recovered from *Eimeria* infection. Immunoblot, analysis showed that rEnGAM22 was recognized not only by the convalescent serum from *E. necatrix*-infected chickens, but also by that from *E. tenella*- and *E. maxima*-infected chickens, respectively. These results suggested that antigens to *E. necatrix* gametocytes, like those of *E. tenella* and *E. maxima*[41,42], might be used to develop a subunit vaccine against avian coccidiosis, and the En*gam22* gene, like Em*gam56* and Em*gam82*, might serve as a novel candidate genes to develop a recombinant subunit vaccine.

## Conclusions

In summary, we cloned a novel gene, En*gam22*, encoding a gametocyte protein from *E. necatrix*, which is an ortholog to Et*gam22* of *E. tenella*. Predictive analysis of this gene sequence revealed a His- and Pro-rich domain. The recombinant protein expressed in a bacterial expression vector had antigenic cross-reactivity to *E. tenella* and *E. maxima*. En*gam22* expression begins in the early macrogametocyte and its native protein is involved in oocyst wall formation in *E. necatrix*.

## Competing interests

The authors declare that they have no competing interests.

## Authors’ contributions

JP and DL conceived and designed the study, and critically revised the manuscript. DL, LC, YZ, CD, SS performed the experiments, analyzed the data and drafted the manuscript. JX, WJ, JL, LW helped in the study design. All authors read and approved the final manuscript.
